# Cholangioscopy-assisted rendezvous method in the bile duct using a 5-Fr nasobiliary tube via an endosonographically created route

**DOI:** 10.1055/a-2973-8445

**Published:** 2026-10-28

**Authors:** Takumi Okuaki, Yusuke Takasaki, Yusuke Yamaguchi, Tomoya Takahashi, Sho Takahashi, Toshio Fujisawa, Hiroyuki Isayama

**Affiliations:** 1 Department of Gastroenterology, Graduate School of Medicine 12847 Juntendo UniversityBunkyoTokyoJapan; 2 Division of Gastroenterology, Department of Medicine 65103 Chulalongkorn University Faculty of MedicineBangkokBangkokThailand

## Abbreviations



**Video 1** The cholangioscopy-assisted rendezvous method in the bile duct using a 5 Fr nasobiliary tube via an endosonographically created route. The video link below:

EUS-BD/A: endoscopic ultrasonography-guided biliary drainage/anastomosis
ERCP: endoscopic retrograde cholangiopancreatography
ESCR: endosonographically created route
POCS: peroral cholangioscopy
CBD: common bile duct
ENBD: endoscopic nasobiliary drainage
PS: plastic stent
GW: guidewire

Passage of the tight biliary strictures with a guidewire is often difficult. Endoscopic ultrasonography-guided biliary drainage/anastomosis (EUS-BD/A) is a rescue procedure when endoscopic retrograde cholangiopancreatography (ERCP) is difficult or fails. Trans-endosonographically created route (ESCR) peroral cholangioscopy (POCS) is useful and safe. 1​ ​2​ We report a cholangioscopy-assisted rendezvous method in the bile duct using a 5-Fr nasobiliary tube (ENBD) via an ESCR ( Video 1​ ). A 74-year-old woman with autosomal dominant polycystic kidney disease developed cholangitis due to a left intrahepatic biliary stricture caused by hepatic cyst compression. Transpapillary drainage failed because the plastic stent (PS) could not pass the stricture due to acute angulation of the bile duct though the guidewire (GW) succeeded in passing. Then, EUS-guided hepaticogastrostomy was performed at B3 with PS. Although contrast medium passed into the common bile duct (CBD), the GW could not pass the stricture. We first attempted to pass the stricture via the ESCR, but the guidewire only reached B4 ( Fig. 1 ). Transpapillary rendezvous failed to approach B3 after passing the stricture. Subsequently, an ENBD tube was placed via the transpapillary route in B4, which was accessible via the ESCR. A cholangioscope (SpyGlass DS II; Boston Scientific, Marlborough, Massachusetts, USA) was advanced through the ESCR into B4, where the ENBD tube was directly visualized, grasped with forceps (SpyBite; Boston Scientific), and pulled into the stomach ( Fig. 2 ). The tube broke off during withdrawal through the endoscope, requiring us to pass a guidewire through it and snare the wire. After trans-ESCR balloon dilation, a PS was placed across the stricture into the CBD. The rendezvous method with POCS in the bile duct was novel and effective with both the transpapillary route and the ESCR. The placement of an ENBD tube may contribute to a successful procedure by improving visibility and stability compared with guidewire rendezvous.

**Fig. 1:**
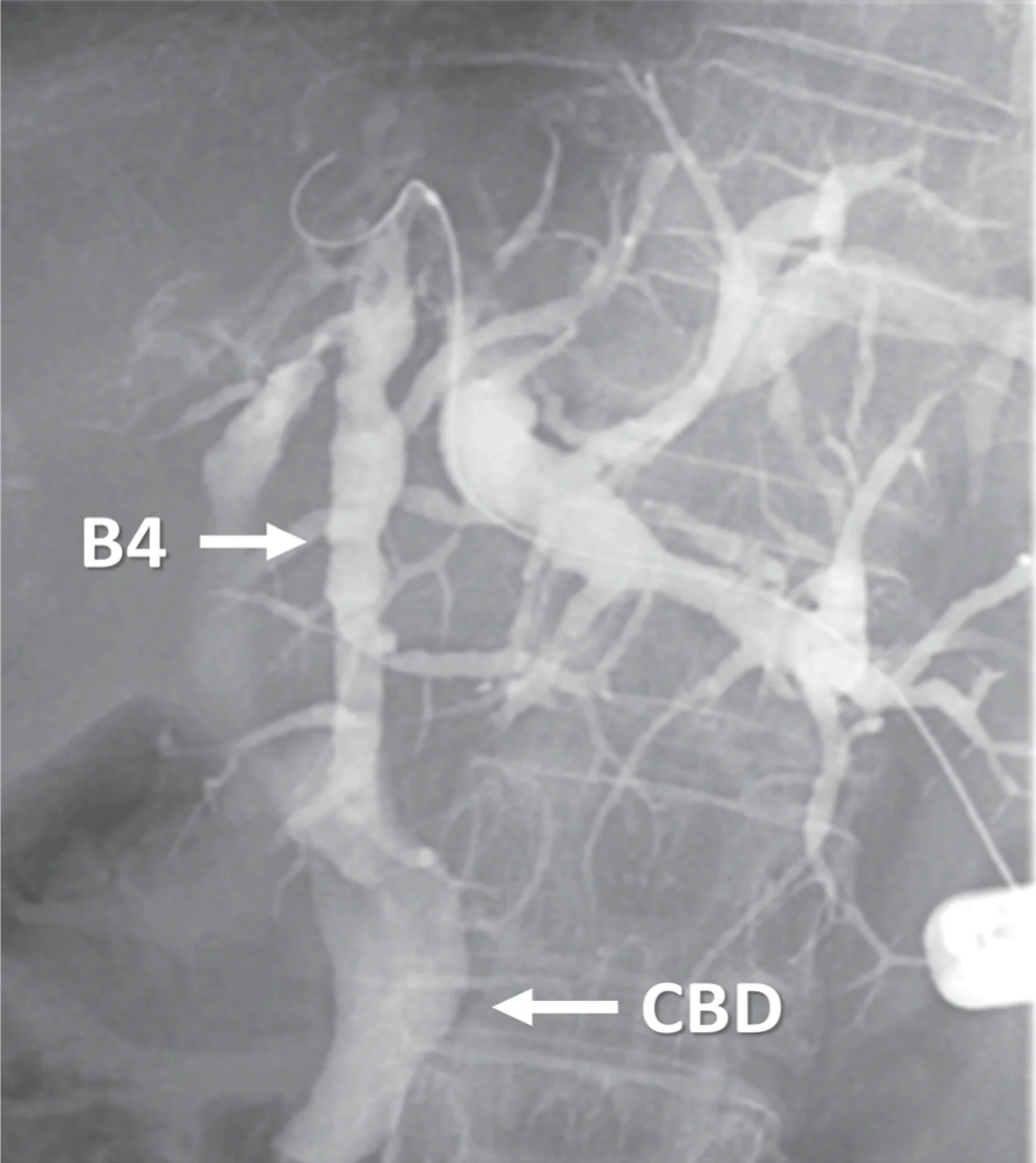
A fluoroscopic image obtained via the endosonographically created route (ESCR). The common bile duct was opacified with contrast medium, but the guidewire could be advanced only into B4.

**Fig. 2:**
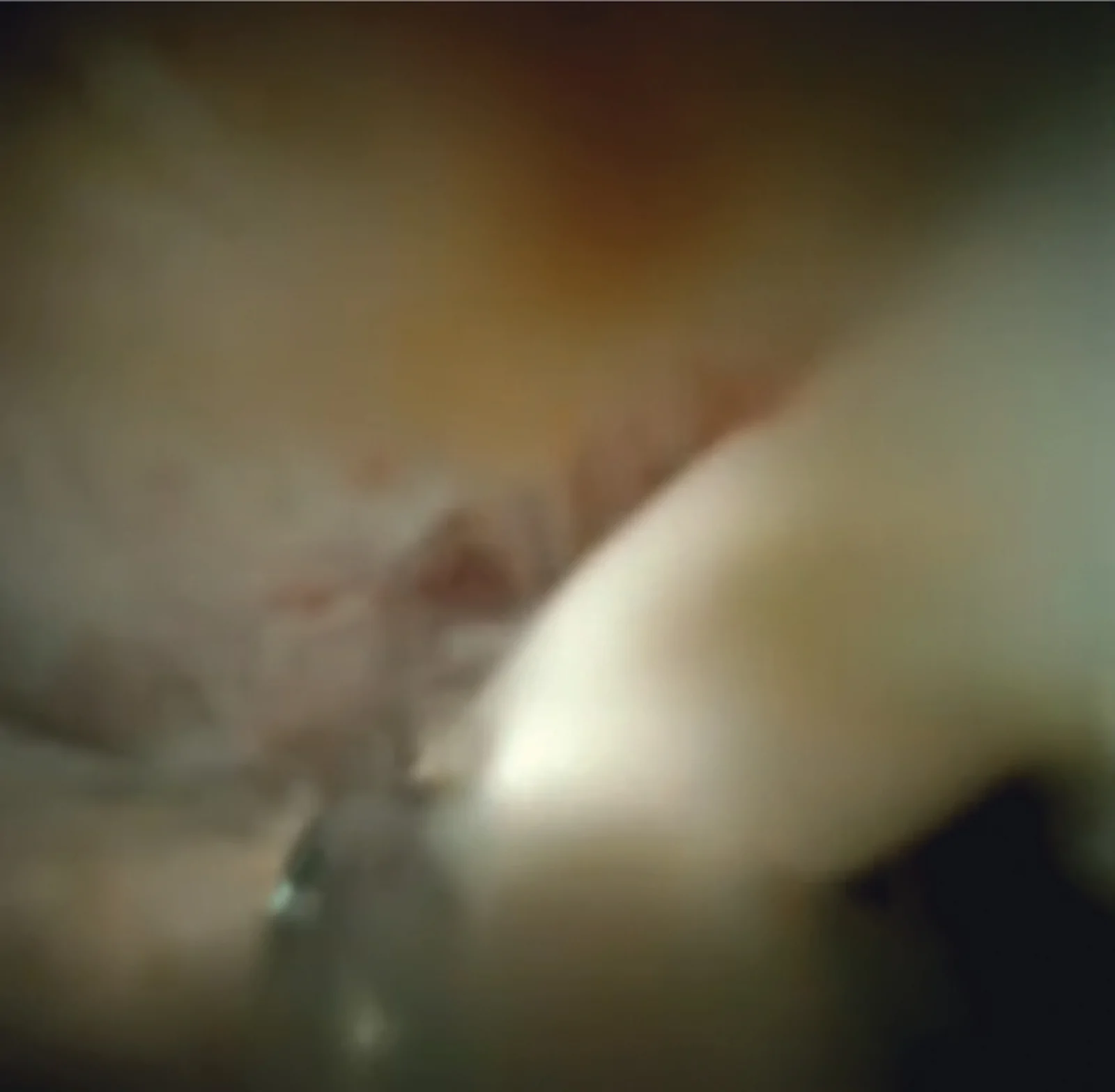
The endoscopic nasobiliary drainage tube placed in B4, visualized through the ESCR with a peroral cholangioscope and grasped with SpyBite forceps.

Endoscopy_UCTN_Code_TTT_1AR_2AG

## Data Availability

All data supporting the findings of this study are included in this article and its accompanying video.

## Declaration of GenAI Use

The authors used Claude (Anthropic) to assist with English language editing of this manuscript. The authors reviewed and edited all contents and take full responsibility for the content of the publication. No generative artificial intelligence was used to create or alter any image or video included in this article.
